# Antibiotics prescription for targeted therapy of pediatric invasive pneumococcal diseases in China: a multicenter retrospective study

**DOI:** 10.1186/s12879-021-06860-8

**Published:** 2021-11-15

**Authors:** Tian-ming Chen, Wen-hui Li, Fang Wang, Kun Tan, Qing-xiong Zhu, Kai Zhou, Shi-hua Liu, Jing Liu, Jing Bi, Hui-ling Deng, Xue-xia Chen, Juan Li, Yu-min Wang, Qing Zhao, Liang Zhu, Hui-xuan Ma, Zhi Li, Ji-kui Deng, Chun-hui Zhu, Kang-kang Wu, Ai-wei Lin, Shuang-jie Li, Dong-meng Wang, Hui-jun Cai, Shi-yong Zhao, Xu-dong Lu, Lan Ye, Fang Dong, Wen-shuang Zhang, Yong-hong Yang, Gang Liu

**Affiliations:** 1 Department of Infectious Diseases, Beijing Children’s Hospital, Capital Medical University, National Center for Children’s Health, Nalishi Road 56#, Xicheng, Beijing, 100045 China; 2 Department of Infection, Hebei Children’s Hospital, Shijiazhuang, China; 3 Infectious Diseases Department, Children’s Hospital Affiliated to Zhengzhou University, Zhengzhou, China; 4 Division of Infectious Diseases, Shenzhen Children’s Hospital, Shenzhen, China; 5 Department of Infectious Disease, Jiangxi Provincial Children’s Hospital, Nanchang, China; 6 Department of Infection, Nanjing Children’s Hospital Affiliated to Nanjing Medical University, Nanjing, China; 7 Department of Infectious Disease, Children’s Hospital of Jinan, Jinan, China; 8 Department of Infection, Hunan Children’s Hospital, Changsha, China; 9 Infection Division, Baoding Children’s Hospital, Baoding, China; 10 Department 2 of infection, Xi’an Children’s Hospital, Xi’an, China; 11 Department of Infectious Disease, Hangzhou Children’s Hospital, Hangzhou, China; 12 Department of Infectious Diseases, Urumqi Children’s Hospital, Urumqi, China; 13 Department of Pediatrics, Maternal and Child Health Care Hospital of Inner Mongolia, Hohhot, China; 14 Department of Infectious Diseases, Children’s Hospital of Shanxi, Taiyuan, China; 15 Department of Hepatology, Hunan Children’s Hospital, Changsha, China; 16 Clinical Laboratory, Xi’an Children’s Hospital, Xi’an, China; 17 Department of Respiratory Medicine, Tianjin Children’s Hospital, Tianjin, China; 18 Beijing Pediatric Research Institute, Beijing Children’s Hospital, Capital Medical University, National Center for Children’s Health, Beijing, China

## Abstract

**Background:**

*Streptococcus pneumoniae* (*S. pneumoniae*) is a major cause of bacterial meningitis, septicemia and pneumonia in children. Inappropriate choice of antibiotic can have important adverse consequences for both the individual and the community. Here, we focused on penicillin/cefotaxime non-susceptibility of *S. pneumoniae* and evaluated appropriateness of targeted antibiotic therapy for children with IPD (invasive pneumococcal diseases) in China.

**Methods:**

A multicenter retrospective study was conducted in 14 hospitals from 13 provinces in China. Antibiotics prescription, clinical features and resistance patterns of IPD cases from January 2012 to December 2017 were collected. Appropriateness of targeted antibiotics therapy was assessed.

**Results:**

806 IPD cases were collected. The non-susceptibility rates of *S. pneumoniae* to penicillin and cefotaxime were 40.9% and 20.7% respectively in 492 non-meningitis cases, whereas those were 73.2% and 43.0% respectively in 314 meningitis cases. Carbapenems were used in 21.3% of non-meningitis cases and 42.0% of meningitis cases for targeted therapy. For 390 non-meningitis cases with isolates susceptible to cefotaxime, vancomycin and linezolid were used in 17.9% and 8.7% of cases respectively for targeted therapy. For 179 meningitis cases with isolates susceptible to cefotaxime, vancomycin and linezolid were prescribed in 55.3% and 15.6% of cases respectively. Overall, inappropriate targeted therapies were identified in 361 (44.8%) of 806 IPD cases, including 232 (28.8%) cases with inappropriate use of carbapenems, 169 (21.0%) cases with inappropriate use of vancomycin and 62 (7.7%) cases with inappropriate use of linezolid.

**Conclusions:**

Antibiotic regimens for IPD definite therapy were often excessive with extensive prescription of carbapenems, vancomycin or linezolid in China. Antimicrobial stewardship programs should be implemented to improve antimicrobial use.

## Introduction

*Streptococcus pneumoniae* is a major cause of bacterial meningitis, septicemia and pneumonia worldwide [1]. With the application of *S. pneumoniae* conjugate vaccine, the incidence of IPD has decreased in developed countries [2]. However, the pneumococcal conjugate vaccine is not universally used in China. *S. pneumoniae* was still the most common pathogen for pneumonia and meningitis in Chinese children under 5 years of age [3, 4].

Unnecessary or inappropriate choice of antibiotics can have important consequences for both the individual and the community, including modification of the enteric microbial ecology and the emergence of antimicrobial resistance. The issuance of guidelines for antibacterial use in clinical practice by the National Health Commission (NHC) of China in October 2004 encouraged more rational use of antibiotics [5]. The guidelines divide antibiotics into non-restricted use (i.e., recommended first-line drugs), restricted use and special use grades. Carbapenems, vancomycin and linezolid, which were often used in therapy of IPD in China [6–8], were classified as special use grades according to the NHC guidelines [5]. Data on appropriateness assessment of antibiotics prescription for IPD in China is rare. In this retrospective study, we focused on penicillin/cefotaxime non-susceptibility of *S. pneumoniae* and evaluated appropriateness of targeted antibiotic therapy for pediatric IPD in China.

## Methods

### Study design and setting

A multicenter retrospective study was conducted in China. Hospitals included in this study should met following criteria: (1) a hospital must have adequate research capabilities to conduct the study, especially laboratory facilities for bacterial culture and the ability to assess susceptibility to antimicrobials; and (2) a hospital must be willing to participate in this research and have enough time to do this study. 14 hospitals (13 tertiary hospitals and 1 secondary hospital) from 13 provinces joined our study. The selected hospitals consisted of 5 in north China, 4 in east China, 3 in south China and 2 in the northwest China (Table 1). Demographic data, clinical features, laboratory findings, antibiotics prescription and clinical outcome of all patients with IPD in these hospitals were collected by local researchers. We considered clinical status on the day of discharge as the clinical outcome.

**Table 1:** Number of cases included in each hospital

Region	Province	Hospital	Study period	Number of cases
North	Beijing	Beijing Children’s Hospital	2012–2017	164
	Hebei	Hebei Children’s Hospital	2012–2017	158
	Hebei	Baoding Children’s Hospital	2012–2017	27
	Inner Mongolia	Maternal and Child Health Care Hospital of Inner Mongolia	2014–2017	10
	Shanxi	Children's Hospital of Shanxi	2015–2017	9
East	Jiangxi	Jiangxi Provincial Children’s Hospital	2012–2017	63
	Jiangsu	Nanjing Children’s Hospital Affiliated to Nanjing Medical University	2012–2017	44
	Zhejiang	Hangzhou Children’s Hospital	2012–2017	22
	Shandong	Children's Hospital of Jinan	2014–2017	32
South	Guangdong	Shenzhen Children’s Hospital	2012–2017	97
	Hunan	Hunan Children's Hospital	2014–2017	28
	Henan	Children's Hospital Affiliated to Zhengzhou University	2015–2017	112
Northwest	Xinjiang	Urumqi Children’s Hospital	2012–2017	15
	Shaanxi	Xi’an Children’s Hospital	2015–2017	25

### Study population

Inclusion criteria: (1) The admission day was between January 1, 2012 and December 31, 2017. For the reason of medical data storage system in different hospitals, we can’t get all data of IPD cases from January 2012 through December 2017 in these 14 hospitals. The study periods for each hospital were shown in Table 1. (2) Hospitalized children younger than 18 years old with diagnosis of IPD in selected hospitals. (3) Clinical data should be available. Invasive pneumococcal infection was defined as illness in which *S. pneumoniae* was isolated from a normally sterile body site. For that PCR or antigen based testing for *S. pneumoniae* was not widely used in china and can’t provide antibiotics susceptibility, we only included patients from whom *S. pneumoniae* was cultured.

Exclusion criteria: (1) Less than two days of targeted antibiotic therapy for IPD. (2) Evidence of co-bacterial infection in IPD case.

### Antimicrobial susceptibility testing

To rule out repeated strains, only one representative strain from each case was included. When both the blood and cerebrospinal fluid (CSF) cultures were positive in a case, we used only the strain from the CSF sample for antimicrobial susceptibility testing. Automated systems, including VITEK2 (bioMérieux) for 12 hospitals and BD Phoenix (Becton Dickinson) for 2 hospitals, were used to assess antibiotics susceptibility. According to the performance standards for antimicrobial susceptibility testing of the Clinical and Laboratory Standards Institute (CLSI) in 2008 [9], we determined the antibiotics susceptibility of *S. pneumoniae* by the minimum inhibition concentration. Breakpoints of penicillin and cefotaxime/ceftriaxone/cefepime vary depending on whether an isolate is from a nonmeningeal or meningeal site. If an *S. pneumoniae* strain was isolated from a patient without meningitis, we defined its penicillin susceptibility by the parenteral nonmeningeal breakpoint (susceptible, ≤ 2.00 mg/l; intermediate, 4.00 mg/L; resistant, ≥ 8.00 mg/l) and cefotaxime/ceftriaxone/cefepime non-meningeal breakpoint (susceptible, ≤ 1.0 mg/L; intermediate, 2.0 mg/L; resistant, ≥ 4.00 mg/L). If an *S. pneumoniae* strain was isolated from a patient with pneumococcal meningitis, we defined its penicillin susceptibility by the parenteral meningeal breakpoint (susceptible, ≤ 0.06 mg/l; resistant, ≥ 0.12 mg/l) and cefotaxime/ceftriaxone/cefepime meningeal breakpoint (susceptible, ≤ 0.50 mg/L; intermediate, 1.00 mg/L; resistant, ≥ 2.00 mg/L). Susceptibility of *S. pneumoniae* to other antibiotics, including clindamycin, erythromycin, trimethoprim-sulfamethoxazole, linezolid, meropenem, vancomycin, levofloxacin, chloramphenicol, amoxicillin and tetracycline, was also determined according to CLSI breakpoints.

### Data of antibiotics prescription

The antibiotics prescription before the culture result was considered as empiric antibiotics therapy. Targeted/definitive antibiotics therapy was defined as antibiotics prescription for treatment of IPD after culture result.

Appropriateness of targeted antibiotics therapy were assessed by two pediatric infectious disease specialists according to 2016 European Society of Clinical Microbiology and Infectious Diseases guideline for diagnosis and treatment of acute bacterial meningitis [10] and Red Book: 2018–2021 Report of the Committee on Infectious Diseases [11]. In this study, we focused on the use of carbapenems, vancomycin and linezolid which are classified as special use grades according to the NHC guidelines and frequently prescribed for IPD targeted therapy in China.

The definitive prescription of carbapenems was considered inappropriate if it met one of following listed criteria: (1) if the organism is nonsusceptible to carbapenems, but carbapenem was still used in targeted therapy; (2) if the organism is susceptible to penicillin or cephalosporins, but carbapenem was still used in targeted therapy without any other reasons, such as hypersensitivity reactions to penicillins or cephalosporins. The definitive prescription of vancomycin or linezolid was considered inappropriate if it met the following criteria: if the organism is susceptible to penicillin or cephalosporins, but vancomycin or linezolid was still used in targeted therapy without any other reasons, such as hypersensitivity reactions to beta-lactam antibiotics.

### Statistical analysis

Categorical variables were presented as numbers and percentages. Continuous variables were presented as median and interquartile range (IQR). Categorical variables were compared using the chi-square or Fisher’s exact tests. Continuous variables were compared by Student’s t test or Mann–Whitney U test according to their distribution. Two-tailed P value of < 0.05 was considered statistically significant. Bonferroni correction was used in multiple comparisons of 4 groups. P value threshold was 0.0083 (0.05/6) when data from 4 regions of China were compared. All statistical analyses were performed with SPSS 17.0 software (IBM Corporation).

## Results

### Demographic data

Overall, 860 IPD cases were collected in 14 hospitals. Forty-nine patients were excluded for less than two days of targeted antibiotic therapy for IPD. Other 5 patients who had evidence of co-bacterial infection were also excluded. Thus, we included 806 children with IPD in our study. The ratio of M/F was 1.56:1. The median age of these patients was 1.3 years (interquartile range 0.8–3.1 years). There was no statistical significance in age and gender in different regions of China (Table 2).

**Table 2:** Characteristics of children presenting with IPD from different regions of China

	All Patients n = 806	North n = 368	East n = 161	South n = 237	Northwest n = 40	p^*^
Median age, years (interquartile range)	1.3 (0.8–3.1)	1.3 (0.7–3.2)	1.6 (0.8–3.5)	1.3 (0.8–2.9)	1.1 (0.7–3.0)	0.287
Male	491 (60.9)	217 (59.0)	113 (70.2)	139 (58.6)	22 (55.0)	0.058
Underlying disease	101 (12.5)	59 (16.0)	25 (15.5)	14 (5.9)	3 (7.5)	0.001
ICU admission	241 (29.9)	124 (33.7)	41 (25.5)	63 (26.6)	13 (32.5)	0.144
Tracheal intubation	112 (13.9)	71 (19.3)	20 (12.4)	17 (7.2)	4 (10.0)	0.000
Death	92 (11.4)	54 (14.7)	18 (11.2)	16 (6.8)	4 (10.0)	0.023
Meningitis	314 (39.0)	145 (39.4)	61 (37.9)	90 (38.0)	18 (45.0)	0.845
Spn not susceptible to penicillin	431 (53.5)	212 (57.6)	64 (39.8)	135(57.0)	20 (50.0)	0.001
Spn not susceptible to cefotaxime	237 (29.4)	127 (34.5)	34 (21.1)	61 (25.7)	15 (37.5)	0.005

Spn *Streptococcus pneumoniae*

^*^All the four regions were compared

### Clinical findings

In our study, 101 (12.5%) cases had an underlying disease or causation (leukemia, 3.0%; trauma or surgery within 1 month before onset, 2.6%; congenital heart disease 2.4%; nephrotic syndrome, 2.1%; immunodeficiency, 1.6%; neuroblastoma, 0.5%; bone marrow transplant, 0.4%). Blood cultures were positive in 456 cases and CSF cultures were positive in 133 cases, while other 121 cases had positive cultures of both blood and CSF. There were 92 cases with positive cultures of other sites (pleural effusion, 70 cases; ascites, 10 cases; joint effusion, 9 cases; bone marrow, 2 cases; subdural effusion, 1 case).

Pneumococcal meningitis was diagnosed in 314 patients. There were more pneumococcal meningitis cases than there were positive CSF cultures because some meningitis cases had positive blood cultures but negative CSF cultures. Among 492 non-meningitis cases, there were 282 cases with bacteremic pneumonia, 176 cases of bacteremia without focal infection and 34 cases with other types of infection (peritonitis, 10 cases; osteoarticular infection, 9 cases; cellulitis, 7 cases; otitis media, 5 cases; infective endocarditis 2 cases; pericarditis, 1 case).

Two hundred and forty-one patients were admitted in ICU and 112 patients needed tracheal intubation. Sixty-two (19.7%) of 314 patients with meningitis died and 57 (18.2%) presented with sequelae (28 with seizures, 8.9%; 20 with hearing loss, 6.4%; 2 with paralysis, 0.6%; and 9 with intellectual disability, 2.9%). Thirty (6.1%) of 492 patients with non-meningitis disease died. There were 4 patients with lung bullae and 2 patients with bronchiectasis in non-meningitis cases. The proportion of patients with underlying disease, tracheal intubation and death differed by regions (all P values < 0.05) (Table 2). The proportion of patients with underlying disease in the north and the east were higher than that in the south and the northwest (all P values < 0.05). The proportion of patients with tracheal intubation was highest in the north(P values < 0.05).

### Antimicrobial susceptibility testing

**Fig. 1:**
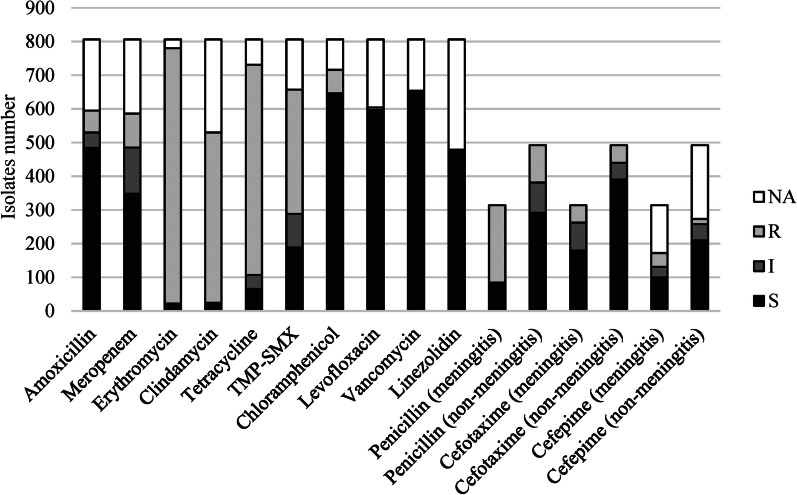
Antibiotic susceptibility of *Streptococcus pneumoniae* isolates identified in the retrospective study (n = 806). *TMP-SMX* trimethoprim-sulfamethoxazole, *S* susceptible, *I* intermediate, *R* resistant, *NA* not analyzed

According to different laboratory facilities of included hospital, different antibiotics were selected for *S. pneumoniae* susceptibility testing. All the isolates from 806 patients underwent penicillin and cefotaxime susceptibility testing. We found 201 (40.9%) *S. pneumoniae* isolates with non-susceptibility to penicillin (90 intermediate and 111 resistant) in the 492 non-meningitis cases. While in the 314 meningitis cases, there were 230 (73.2%) *S. pneumoniae* isolates resistant to penicillin. There are no CLSI criteria for intermediate and resistant about vancomycin and linezolid. All isolates in our study, which got susceptibility test for vancomycin or linezolid, were susceptible to vancomycin with MIC ≤ 0.5ug/ml and also linezolid with MIC ≤ 1ug/ml. Susceptibility for different antibiotics were shown in Fig. 1.

The penicillin non-susceptibility rate of *S. pneumoniae* in IPD cases differed by region (57.6% in the north, 57.0% in the south, 50.0% in the northwest, 39.8% in the east) (Table 2) and penicillin non-susceptibility rate in the north region and south region were higher than that in the east region (north 57.6% vs east 39.8%, p = 0.001; south 57.0% vs east 39.8%, p = 0.001). The cefotaxime non-susceptibility rate also differed by region and cefotaxime non-susceptibility rate in the north region were higher than that in the east region (north 34.5% vs east 21.1%, p = 0.002).

The resistance rates of *S. pneumoniae* to meropenem, erythromycin, clindamycin, tetracycline, sulfamethoxazole, chloramphenicol and levofloxacin were 40.6% (238/586), 97.4% (760/780), 96.0% (509/530), 91.1% (666/731), 71.4% (657/469), 9.9% (71/716) and 2.3% (14/604) respectively. No *S. pneumoniae* isolates were resistant to vancomycin and linezolid (Fig. 1).

### Antibiotics prescription

#### Empiric antibiotics therapy

Twenty-five patients, who had antibiotics susceptibility of *S. pneumoniae* before being hospitalized, got targeted antibiotics therapy directly. Thus, 781 patients with IPD got empiric antibiotics therapy, including penicillins monotherapy (64, 8.2%), cephalosporins monotherapy (427, 54.7%), carbapenem monotherapy (90, 11.5%), cephalosporin plus vancomycin (52, 6.7%), carbapenem plus vancomycin (122, 15.6%), cephalosporin plus linezolid (5, 0.6%), carbapenem plus linezolid (7, 0.9%), cephalosporin plus teicoplanin (3, 0.4%) and macrolides monotherapy (11, 1.4%) (Table 3). The empiric antibiotics prescription did not present significant differences between regions with the exception of penicillin. Penicillin monotherapy was the most commonly used for empiric therapy of IPD in the east region, compared with other regions (Table 3).

**Table 3:** Empiric antibiotics therapy of 781 patients with IPD from different regions of China

	All Patients n = 781	North n = 349	East n = 158	South n = 236	Northwest n = 38	p^*^
Penicillins monotherapy	64 (8.2)	18 (5.2)	23 (14.6)	21 (8.9)	2 (5.3)	0.006
Cephalosporin monotherapy	427 (54.7)	199 (57.0)	78 (49.4)	131 (55.5)	19 (50.0)	0.396
Carbapenem monotherapy	90 (11.5)	43 (12.3)	17 (10.8)	25 (10.6)	5 (13.2)	0.895
Cephalosporin plus vancomycin	52 (6.7)	25 (7.2)	7 (4.4)	18 (7.6)	2 (5.3)	0.566
Carbapenem plus vancomycin	122 (15.6)	47 (13.5)	29 (18.4)	36 (15.3)	10 (26.3)	0.142
Cephalosporin plus linezolid	5 (0.6)	4 (1.1)	0 (0)	1 (0.4)	0 (0)	0.278
Carbapenem plus linezolid	7 (0.9)	5 (1.4)	1 (0.6)	1 (0.4)	0 (0)	0.475
Cephalosporin plus teicoplanin	3 (0.4)	3 (0.9)	0 (0)	0 (0)	0 (0)	0.183
Macrolides monotherapy	11 (1.4)	5 (1.4)	3 (1.9)	3 (1.3)	0 (0)	0.715

^*^All the four regions of China were compared

#### Definitive antibiotics therapy

**Fig. 2:**
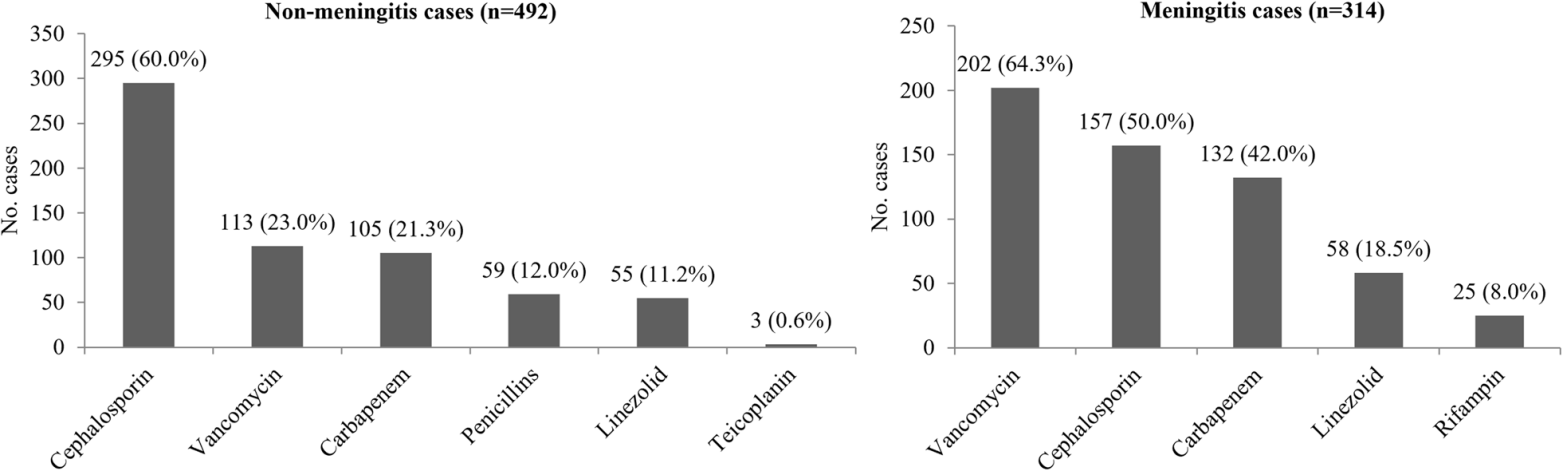
Antibiotics used in definitive therapy in invasive pneumococcal diseases (meningitis and non-meningitis cases)

**Fig. 3:**
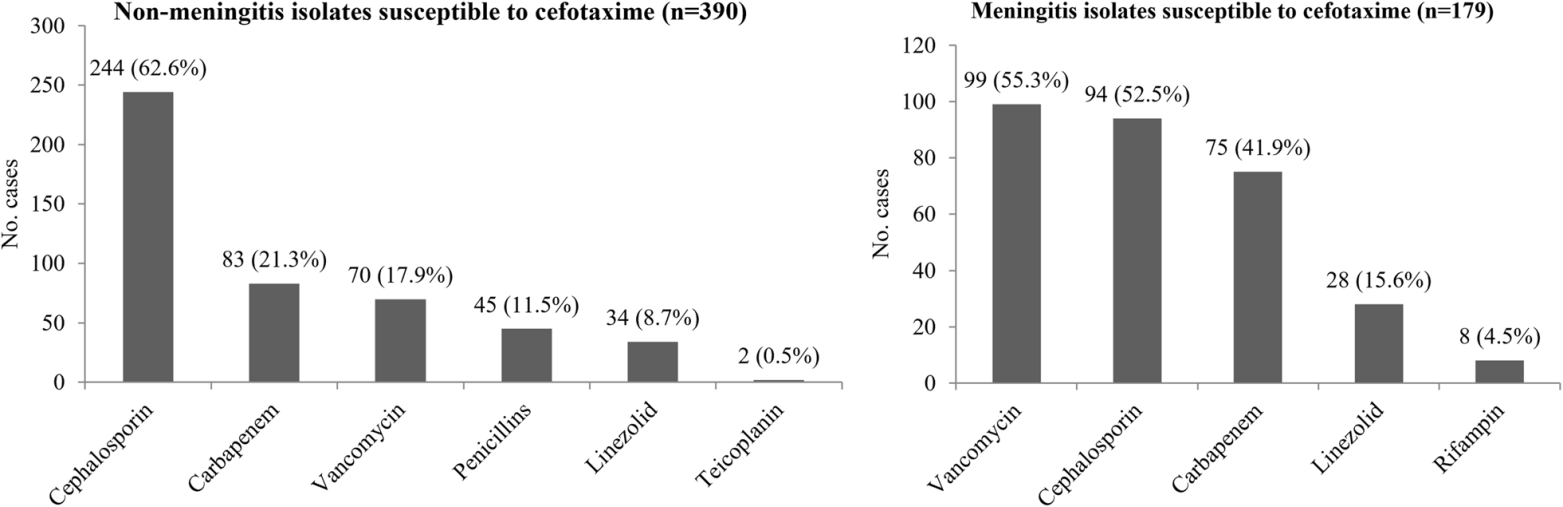
Antibiotics used in definitive therapy in invasive pneumococcal diseases (meningitis and non-meningitis cases) whose isolates were susceptible to cefotaxime

Definitive antibiotics therapy strategy in non-meningitis cases included penicillins monotherapy (54, 11.0%), cephalosporin monotherapy (220, 44.7%), carbapenem monotherapy (47, 9.6%), cephalosporin plus vancomycin (46, 9.3%), carbapenem plus vancomycin (46, 9.3%), cephalosporin plus linezolid (28, 5.7%), carbapenem plus linezolid (12, 2.4%), vancomycin monotherapy (18, 3.7%), linezolid monotherapy (13, 2.6%) and other prescriptions (8, 1.6%) (Table 4). Thus, cephalosporin was prescribed in 295 (60.0%) cases and was most common antibiotic used in non-meningitis cases, followed by vancomycin (113 cases, 23.0%), carbapenems (105 cases, 21.3%), penicillin (59 cases, 12.0%), linezolid (55 cases, 11.2%) and teicoplanin (3 cases, 0.6%) (Fig. 2). Among 390 non-meningitis cases whose isolates were susceptible to cefotaxime, carbapenems, vancomycin and linezolid were prescribed in 83 (21.3%) cases, 70 (17.9%) cases and 34 (8.7%) cases respectively (Fig. 3).

**Table 4:** Appropriateness of antibiotic prescription for targeted therapy of non-meningitis IPD

Definitive antibiotics therapy	All patients n = 492	Spn susceptible to penicillin n = 291	Spn not susceptible to penicillin n = 201			p
			Susceptible to cefotaxime n = 99	Not susceptible to cefotaxime n = 102	Subtotal n = 201	
Penicillins monotherapy	54 (11.0)	29 (10.0)	12 (12.1)	13 (12.7)	25 (12.4)	0.389
Cephalosporin monotherapy	220 (44.7)	155 (53.3)	47 (47.5)	18 (17.6)	65 (32.3)	0.000
Carbapenem monotherapy	47 (9.6)	36 (12.4)	5 (5.1)	6 (2.0)	11 (5.5)	0.011
Cephalosporin plus vancomycin	46(9.3)	17 (5.8)	8 (8.1)	21 (20.6)	29 (14.4)	0.001
Carbapenem plus vancomycin	46 (9.3)	20 (6.9)	12 (12.1)	14 (13.7)	26 (12.9)	0.023
Cephalosporin plus linezolid	28 (5.7)	13 (4.5)	3 (3.0)	12 (11.8)	15 (7.5)	0.159
Carbapenem plus linezolid	12 (2.4)	5 (1.7)	5 (5.1)	2 (2.0)	7 (3.5)	0.342
Vancomycin monotherapy	18 (3.7)	5 (1.7)	5 (5.1)	8 (7.8)	13 (6.5)	0.006
Linezolid monotherapy	13 (2.6)	5 (1.7)	2 (2.0)	6 (5.9)	8 (4.0)	0.124
Other prescriptions	8 (1.6)	6^a^ (2.1)	0 (0)	2^b^ (2.0)	2 (1.0)	–
Inappropriate use of						
Carbapenems	99 (20.1)	61 (21.0)	22 (22.2)	16 (15.7)	38 (18.9)	0.576
Vancomycin	70 (14.2)	45 (15.5)	25 (25.3)	0 (0)	25 (12.4)	0.345
Linezolid	34 (6.9)	24 (8.2)	10 (10.1)	0 (0)	10 (5.0)	0.160
No. patients with inappropriate antibiotic use	161 (32.7)	105 (36.1)	40 (40.4)	16 (15.7)	56 (27.9)	0.056

Spn, *Streptococcus pneumoniae*

^a^2 patients with penicillins plus vancomycin, 1 patient with penicillins plus linezolid, 1 patient with Cephalosporin plus teicoplanin and 1 patient with teicoplanin monotherapy

^b^1 patient with penicillins plus linezolid and 1 patient with teicoplanin monotherapy

Definitive antibiotics therapy strategy in meningitis cases included cephalosporin monotherapy (39, 12.4%), carbapenem monotherapy (15, 4.8%), cephalosporin plus vancomycin (88, 28.0%), carbapenem plus vancomycin (94, 29.9%), cephalosporin plus linezolid (26, 8.3%), carbapenem plus linezolid (21, 6.7%), vancomycin plus rifampin (16, 5.1%), linezolid monotherapy (6, 1.9%) and other prescriptions (9, 1.6%) (Table 5). The most common antibiotic used in meningitis cases was vancomycin (202 cases, 64.3%), followed by cephalosporin (157 cases, 50.0%), carbapenems (132 cases, 42.0%), linezolid (58 cases, 18.5%) and rifampin (25 cases, 8.0%) (Fig. 2). Among 179 meningitis cases whose isolates were susceptible to cefotaxime, vancomycin carbapenems, and linezolid were prescribed in 99 (55.3%) cases, 75 (41.9%) cases and 28 (15.6%) cases respectively (Fig. 3).

**Table 5:** Appropriateness of antibiotic prescription for targeted therapy of meningitis IPD

Definitive antibiotics therapy	All patients n = 314	Spn susceptible to penicillin n = 84	Spn not susceptible to penicillin n = 230			p
			Susceptible to cefotaxime n = 95	Not susceptible to cefotaxime n = 135	Subtotal n = 230	
Penicillins monotherapy	0 (0)	0 (0)	0 (0)	0 (0)	0 (0)	–
Cephalosporin monotherapy	39 (12.4)	19 (22.6)	18 (18.9)	2 (1.5)	20 (8.7)	0.001
Carbapenem monotherapy	15 (4.8)	9 (10.7)	6 (6.3)	0 (0)	6 (2.6)	0.007
Cephalosporin plus vancomycin	88 (28.0)	18 (21.4)	26 (27.4)	44 (32.6)	70 (30.4)	0.116
Carbapenem plus vancomycin	94(29.9)	21 (25.0)	26 (27.4)	47 (34.8)	73 (31.7)	0.248
Cephalosporin plus linezolid	26 (8.3)	5 (6.0)	8 (8.4)	13 (9.6)	21 (9.1)	0.366
Carbapenem plus linezolid	21 (6.7)	5 (6.0)	7 (7.4)	9 (6.7)	16 (7.0)	0.753
Vancomycin plus rifampin	16 (5.1)	5 (6.0)	2 (2.1)	9 (6.7)	11 (4.8)	0.899
Vancomycin monotherapy	0 (0)	0 (0)	0 (0)	0 (0)	0 (0)	–
Linezolid monotherapy	6 (1.9)	2 (2.4)	1 (1.1)	3 (2.2)	4 (1.7)	1.000
Other prescriptions	9 (2.9)	0 (0)	1^a^ (1.1)	8^b^ (5.9)	9 (3.9)	–
Inappropriate use of						
Carbapenems	133 (42.4)	35 (41.7)	40 (42.1)	58 (43.0)	98 (42.6)	0.881
Vancomycin	109 (34.7)	54 (64.3)	55 (57.9)	0 (0)	55 (23.9)	0.000
Linezolid	28 (8.9)	12 (14.3)	16 (16.8)	0 (0)	16 (7.0)	0.044
No. patients with inappropriate antibiotic use	200 (63.7)	65 (77.4)	77 (81.1)	58 (43.0)	135 (58.7)	0.002

Spn, *Streptococcus pneumoniae*

^a^Carbapenem plus vancomycin and rifampin

^b^2 patients with cephalosporin plus vancomycin and rifampin, 2 patients with cephalosporin plus linezolid and rifampin, 2 patients with linezolid plus rifampin, 1 patient with carbapenem plus vancomycin and rifampin and 1 patient with carbapenem plus linezolid and rifampin,

#### Appropriateness of definitive antibiotics therapy

According to the aforementioned selected appropriateness criteria for use of carbapenems, vancomycin and linezolid, inappropriate therapies were identified in 361 (44.8%) out of 806, including 232 (28.8%) cases with inappropriate use of carbapenems, 169 (21.0%) cases with inappropriate use of vancomycin and 62 (7.7%) cases with inappropriate use of linezolid. There was inappropriate use of carbapenems, vancomycin or linezolid in 161 (32.7%) non-meningitis cases, including 99 (20.1%) cases with inappropriate use of carbapenems, 70 (14.2%) cases with inappropriate use of vancomycin and 34 (6.9%) cases with inappropriate use of linezolid (Table 4). Inappropriate use of carbapenems, vancomycin or linezolid was found in 200 (63.7%) meningitis cases, including 133 (42.4%) cases with inappropriate use of carbapenems, 109 (34.7%) cases with inappropriate use of vancomycin and 28 (8.9%) cases with inappropriate use of linezolid (Table 5).

Inappropriate antibiotics therapy group of non-meningitis had higher proportion of patients with underlying disease, ICU admission, tracheal intubation and death (all P values < 0.02), compared with appropriate antibiotics therapy group of non-meningitis (Table 6). Similarly, inappropriate antibiotics therapy group of meningitis also had higher proportion of patients with ICU admission, tracheal intubation and death (all P values < 0.001), compared with appropriate antibiotics therapy group of meningitis (Table 6).

**Table 6:** Baseline characteristics of the patients with IPD (meningitis cases and non-meningitis cases) receiving appropriate/inappropriate antibiotic prescription

	Meningitis cases n = 314			Non-meningitis cases n = 492		
	Appropriate therapy n = 114	Inappropriate therapy n = 200	p	Appropriate therapy n = 331	Inappropriate therapy n = 161	p
Median age, years (interquartile range)	1.1 (0.6–3.6)	1.1 (0.5–3.2)	0.733	1.5 (0.9–3.1)	1.8 (1.0–3.1)	0.075
Male	69 (60.5)	123 (61.5)	0.865	202 (61.0)	97 (60.2)	0.868
Underlying disease	7 (6.1)	23 (11.5)	0.120	27 (8.2)	44 (27.3)	0.000
ICU admission	35 (30.7)	108 (54.0)	0.000	41 (12.4)	57 (35.4)	0.000
Tracheal intubation	9 (7.9)	59 (29.5)	0.000	14 (4.2)	30 (18.6)	0.000
Death	9 (7.9)	53 (26.5)	0.000	14 (4.2)	16 (9.9)	0.013
Spn not susceptible to penicillin	95 (83.3)	135 (67.5)	0.002	125 (60.5)	56 (32.3)	0.056

Inappropriate use of carbapenems (42.2% in the east, 30.0% in the northwest, 26.2% in the south, 24.5% in the north), vancomycin (31.7% in the east, 19.8% in the north, 16.9% in the south, 12.5% in the northwest) and linezolid (11.1% in the north, 7.2% in the south, 1.9% in the east, 2.5% in the northwest) all differed by region (all P values < 0.02) (Table 7). Inappropriate use rate of carbapenems and vancomycin was highest in the east. Nevertheless, inappropriate use rates of linezolid were higher in the north and the south than that in the east and the northwest (Table 7).

**Table 7:** Appropriateness of antibiotic prescription for targeted therapy of IPD from different regions of China

	All Patients n = 806	North n = 368	East n = 161	South n = 237	Northwest n = 40	p^*^
Penicillins monotherapy	54 (6.7)	20 (5.4)	20 (12.4)	13 (5.5)	1 (2.5)	0.019
Cephalosporin monotherapy	259 (32.1)	96 (26.1)	44 (27.3)	102 (43.0)	17 (42.5)	0.000
Carbapenem monotherapy	62 (7.7)	24 (6.5)	16 (9.9)	19 (8.0)	3 (7.5)	0.607
Cephalosporin plus vancomycin	134 (16.6)	72 (19.6)	20 (12.4)	33 (13.9)	9 (22.5)	0.086
Carbapenem plus vancomycin	140 (17.4)	51 (13.9)	49 (30.4)	31 (13.1)	9 (22.5)	0.000
Cephalosporin plus linezolid	54 (6.7)	38 (10.3)	1 (0.6)	15 (6.3)	0 (0)	0.000
Carbapenem plus linezolid	33 (4.1)	15 (4.1)	3 (1.9)	14 (5.9)	1 (2.5)	0.204
Vancomycin plus rifampin	16 (2.0)	13 (3.5)	1 (0.6)	2 (0.8)	0 (0)	0.038
Vancomycin monotherapy	18 (2.2)	9 (2.4)	3 (1.9)	6 (2.5)	0 (0)	0.556
Linezolid monotherapy	19 (2.4)	17 (4.6)	1 (0.6)	1 (0.4)	0 (0)	0.001
Other prescriptions	17 (2.1)	13^a^ (3.5)	3^b^ (1.9)	1^c^ (0.4)	0 (0)	–
Inappropriate use of						
Carbapenems	232 (28.8)	90 (24.5)	68 (42.2)	62 (26.2)	12 (30.0)	0.000
Vancomycin	169 (21.0)	73 (19.8)	51 (31.7)	40 (16.9)	5 (12.5)	0.001
Linezolid	62 (7.7)	41 (11.1)	3 (1.9)	17 (7.2)	1 (2.5)	0.000
No. patients with inappropriate antibiotic use	361 (44.8)	169 (45.9)	88 (54.7)	90 (38.0)	14 (35.0)	0.006

^a^2 patients with cephalosporin plus vancomycin and rifampin, 2 patients with cephalosporin plus linezolid and rifampin, 2 patients with linezolid plus rifampin, 2 patients with carbapenem plus vancomycin and rifampin, 2 patients with teicoplanin monotherapy, 1 patient with carbapenem plus linezolid and rifampin, 1 patient with penicillins plus linezolid and 1 patient with Cephalosporin plus teicoplanin

^b^3 patients with penicillins plus vancomycin,

^c^1 patient with penicillins plus linezolid

^*^All the four regions were compared

## Discussion

Our study results highlight the problem of antibiotics use in the management of IPD in China. To our knowledge, this is the first multicenter study about appropriateness of antibiotic therapy for IPD in China. The retrospective study results revealed that up to 44.8% of the prescriptions were inappropriate and excessive. The inappropriate use of the carbapenems, vancomycin and linezolid in *S. pneumoniae* meningitis was even higher at 63.7%. Clinicians should be retrained on the management of IPD, especially meningitis, and de-escalate antibiotics for targeted therapy.

We observed many improper uses of carbapenems: 42.4% in meningitis cases and 20.1% in non-meningitis cases. According to the clinical application evaluation rules of carbapenems released by the NHC in 2018 [12], carbapenems should be reserved for severe infection caused by aerobic gram-negative bacilli with multiple drug resistance, severe mixed infection of aerobic bacteria and anaerobic bacteria such as *Bacteroides fragilis* and empiric treatment of infection in patients with severe immunodeficiency before pathogen identified. Carbapenems use was often unnecessary in definitive antibiotics therapy for IPD unless other pathogens were detected for reasonable use. The spread of carbapenem-resistant Gram-negative bacteria (GNB) with the consequent change in institutional epidemiology continues to evolve rapidly worldwide [13]. Previous studies have showed that carbapenem resistance in GNB appeared to correlate with previous exposure to carbapenems [14–17] while reduction of carbapenem use, the incidence of *Clostridioides difficile* infection decreased [18]. Improper carbapenems use in targeted therapy for IPD should be reduced in China.

Vancomycin was used improperly in 34.7% of meningitis cases and 14.2% of non-meningitis cases, while inappropriate use of linezolid was found in 8.9% of meningitis cases and 6.9% of non-meningitis cases. According to guidelines, vancomycin and linezolid should be used in IPD patients with isolates not susceptible to β-lactams or patients allergic to β-lactams. Unnecessary use of vancomycin and linezolid can result in the emergence of antimicrobial resistance. Previous vancomycin use was a risk factor for vancomycin resistant enterococci (VRE) colonization which increased the risk of subsequent VRE infection [19]. Limiting empiric vancomycin exposure was associated with a decreased incidence of VRE [20]. A relationship was also found between appropriate linezolid use and the incidence of linezolid-resistant strains of *E. faecium, S. epidermidis* and *S. haemolyticus* [21]. To reduce improper use of vancomycin and linezolid for patients with IPD in China, we should educate the clinicians that β-lactams rather than vancomycin or linezolid usually should be the first choice for treatment of *S. pneumoniae* susceptible to penicillins and cephalosporins.

In the present study, patients who get inappropriate use of vancomycin, linezolid or carbapenems in definite therapy had higher proportions of ICU admission, tracheal intubation and death. It means that more severe cases of IPD were prone to get excessive use of antibiotics such as vancomycin, linezolid and carbapenems. Clinicians used the antibiotics in IPD not only according the isolate susceptibility but also by judging the severity of patient. The confounder could be that these patients had an unclear focus of infection so unnecessarily broad spectrum agents and also vancomycin or linezolid, which can always cover majority of gram positive cocci, were continued. This situation looks like reasonable. But all patients in our study had definite diagnosis of IPD and patients with other bacteria co-infection were excluded. Thus, severity of illness in our case series should not be the reason for excessive use vancomycin, linezolid or carbapenems. Clinicians should modify antibiotics according to susceptibility testing results for definite therapy if there is no evidence of other bacteria co-infection.

Our study also showed that improper use rates of carbapenems, vancomycin or linezolid in definitive therapy differed by regions, although empiric antibiotics prescription had little difference. Inappropriate use rates of carbapenems and vancomycin were highest in the east, while inappropriate use rates of linezolid were higher in the north and the south. It seems that clinicians’ compliance with the IPD guidelines was different in regions of China. Education of IPD guidelines should be implemented across China.

The non-susceptibility rates of *S. pneumoniae* to penicillin and cefotaxime were up to 73.2% and 43.0% respectively in meningitis cases. According to guideline [10], cefotaxime or ceftriaxone plus vancomycin or rifampicin should be used empirically for meningitis in area where *S. pneumoniae* antimicrobial sensitivity to penicillin/cefotaxime was reduced. This is the reason for the frequent use of vancomycin and linezolid in empiric therapy in patients with IPD in China. On the other hand, there were higher proportion of underlying disease in the north and east China. It might be reasonable for the clinicians using more carbapenems, vancomycin and linezolid empirically in these regions. We recommend exploring other methods to determine cefotaxime non-susceptibility more rapidly in order to reduce vancomycin and linezolid use empirically in China.

Our study has some limitations. First, this was a retrospective study and we included 13 tertiary hospitals, but only 1 secondary hospital and no primary hospitals. There could be patient selection bias for that more seriously ill children were referred to tertiary-level care than primary and secondary hospital. Also, some participating centers did not involve all the IPD patients through January 1, 2012 to December 31, 2017. The data is not generalizable to the whole of China. However, our study is the largest study of IPD antibiotic treatments in children. Second, the failure to collect the antibiotic dosage of definite therapy in this study might underestimate the inappropriate antibiotics use.

Through this national hospital-based survey, we found that antibiotic regimens for IPD definite therapy were often excessive with extensive prescription of carbapenems, vancomycin or linezolid. There is an urgent need for specific guidelines for IPD in China. Antimicrobial stewardship programs should be implemented to improve antimicrobial use.

## Data Availability

The datasets collected and/or analysed during the current study are available from the corresponding author on reasonable request.

## Abbreviations

*S. pneumoniae*: *Streptococcus pneumonia*
IPD: Invasive pneumococcal diseases
NHC: National Health Commission
CSF: Cerebrospinal fluid
VRE: Vancomycin resistant enterococci
